# Supplementary material for: Cocaine induces locomotor sensitization through a dopamine-dependent VTA-mPFC-FrA cortico-cortical pathway in male mice
Source: Nat Commun. 2023 Mar 21;14:1568. doi: 10.1038/s41467-023-37045-3 (PMC10030897; doi:10.1038/s41467-023-37045-3)
Supplement: Supplementary file 1 — Supplementary Information [file 41467_2023_37045_MOESM1_ESM.pdf]

Supplementary Information

Cocaine induces locomotor sensitization through a dopamine-dependent VTA-mPFC-FrA cortico-cortical pathway in male mice

Lun Wang[1,2,3#], Min Gao[1,2,3#], Qinglong Wang[1,2,3#], Liyuan Sun[1,2,3], Muhammad Younus[1,2,3], Sixing Ma[1,2,3], Can Liu[1,2,3], Li Shi[1,2,3], Yang Lu[1,2,3], Bo Zhou[1,2,3], Suhua Sun[1,2,3], Guoqing Chen[1,2,3], Jie Li[1,2,3], Quanfeng Zhang[1,2,3], Feipeng Zhu[1,2,3*] , Changhe Wang[4,5*], and Zhuan Zhou[1,2,3*]

[1]State Key Laboratory of Membrane Biology and Beijing Key Laboratory of Cardiometabolic Molecular Medicine, Institute of Molecular Medicine, College of Future Technology, Peking University, Beijing 100871, China; [2]Peking-Tsinghua Center for Life Sciences, Peking University, Beijing 100871, China; [3]PKU-IDG/McGovern Institute for Brain Research, Peking University, Beijing 100871, China; [4]Neuroscience Research Center, Institute of Mitochondrial Biology and Medicine, Key Laboratory of Biomedical Information Engineering of Ministry of Education, School of Life Science and Technology, Xi'an Jiaotong University, Xi'an 710049, China; [5]Department of Neurology, the First Affiliated Hospital of Xi'an Jiaotong University, Xi'an 710061, China

[#]These authors contributed equally

[*]Corresponding authors （ZZ, zzhou@pku.edu.cn； CHW, changhecool@163.com； FPZ, flysummer2006@126.com ）

Address for editorial correspondence and proof:
Dr. Zhuan Zhou
Institute of Molecular Medicine,
Peking University
5 Yiheyuan Road,
Beijing 100871, China
Tel & Fax: ++86-10-6275-3212
Email: zzhou@pku.edu.cn
https://future.pku.edu.cn/en/

Supplementary Information

Supplementary information contains supplementary figures S1–S13 and tables S1-S2.

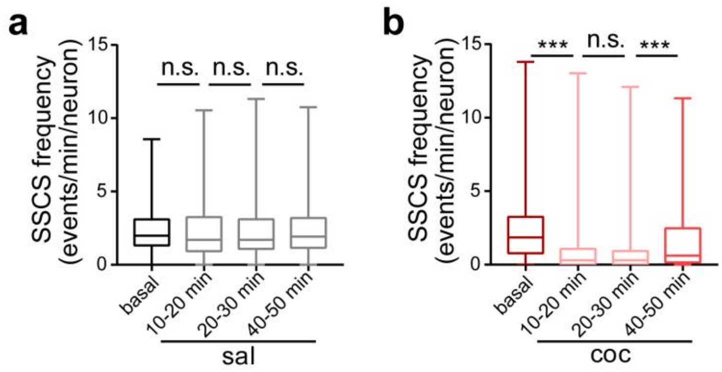

Fig. S1 (related to Fig. 1) Reversible inhibitory effect of cocaine on the FrA. (**a**) Box-and-whisker plot (minimum, maximum and 3 quartiles) of neuronal SSCSs recorded by Cal-520 during 10–20 min, 20–30 min, and 40–50 min after i.p. saline. n = 328 neurons from 4 mice, two-tailed Wilcoxon test. (**b**) As in (**a**) but with cocaine injection. n = 313 neurons from 4 mice, p <0.0001, = 0.95, <0.0001, two-tailed Wilcoxon test. ***p <0.001. Source data are provided as a Source Data file.

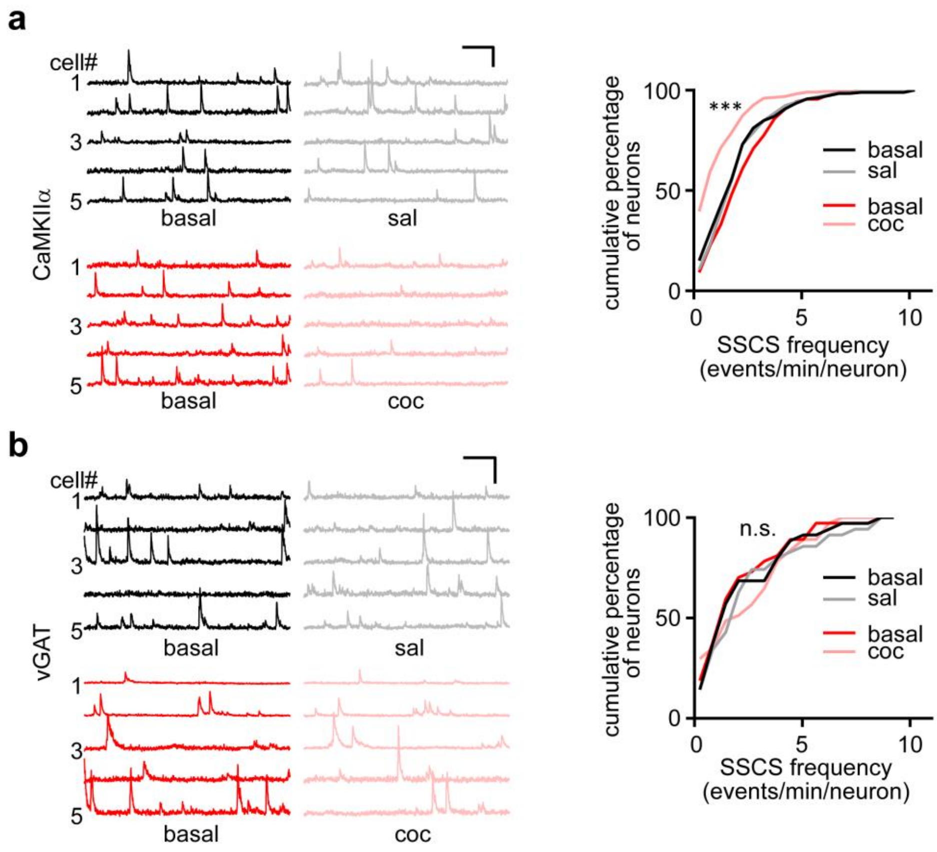

Fig. S2 (related to Fig. 1) Representative SSCS traces and cumulative activity distributions of excitatory and inhibitory FrA neurons. (**a**) Representative SSCS traces and cumulative distribution of excitatory neurons under different conditions. Sal, n = 185 neurons from 4 mice; coc, n = 227 neurons from 5 mice. ***p <0.001, two-tailed Wilcoxon test. Scale bars, 20 s, 200% dF/F_0. (**b**) As in (**a**), except that the SSCS signals were recorded from GABAergic neurons. Sal, n = 35 neurons from 4 mice; coc, n = 37 neurons from 4 mice. n.s., not significant, two-tailed Wilcoxon test. Scale bars, 20 s, 400% dF/F_0. Source data are provided as a Source Data file.

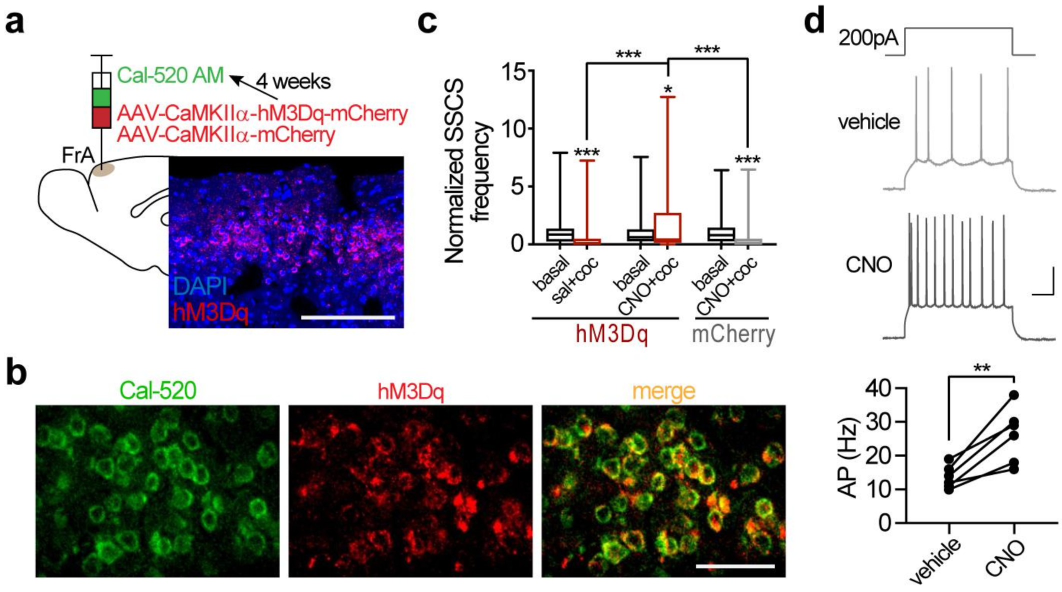

Fig. S3 (related to Fig. 1) Verification of chemogenetic activation of FrA neurons. (**a**) Neuronal Ca^{2+} imaging of the FrA with hM3Dq-mCherry or mCherry expressed in excitatory neurons through AAV-CaMKIIα-hM3Dq-mCherry or AAV-CaMKIIα-mCherry injection. Inset micrograph shows virus expression in a coronal FrA slice with DAPI nuclear staining (blue); scale bar, 200 µm. (**b**) Representative two-photon image of FrA neurons labeled with hM3Dq-mCherry (red) and Cal-520 (green). Scale bar, 50 µm. (**c**) Normalized SSCS frequency (minimum, maximum and 3 quartiles) of FrA excitatory neurons 10–20 min after i.p. cocaine (10 mg/kg). Cocaine was injected 30 min after CNO (0.5 mg/kg, i.p.) or saline injection. hM3Dq: Sal, n = 258 neurons from 4 mice; CNO, n = 228 neurons from 4 mice. mCherry: n = 232 neurons from 4 mice. Two-tailed Wilcoxon test for paired comparisons: p <0.0001, = 0.023, <0.0001; and two-tailed Mann-Whitney test for unpaired comparisons: p <0.0001, 0.0001. (**d**) Upper, representative traces of action potentials (APs) evoked by current injection (200 pA, 500 ms). Lower, averaged firing frequency of hM3Dq-mCherry^{+} FrA neurons in the absence or presence of CNO (10 µM). Scale bars, 100 ms, 30 mV. n = 6 cells from 2

mice. $p = 0.0051$, paired two-tailed *t*-test. *p <0.05, **p <0.01, ***p <0.001. Source data are provided as a Source Data file.

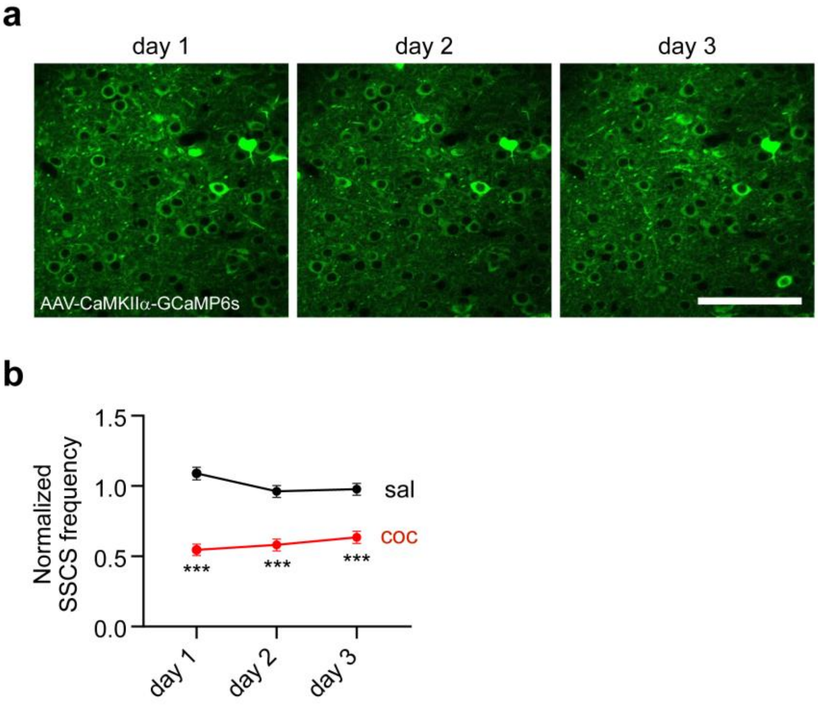

Fig. S4 (related to Fig. 1) Cocaine inhibits excitatory FrA neurons on consecutive days. (**a**) Representative micrographs showing repeated Ca^{2+} imaging of the same FrA excitatory neurons in an awake mouse with AAV-CaMKIIα-GCaMP6s injection. Scale bar, 100 µm. (**b**) Statistics of normalized SSCS frequency after i.p. saline or cocaine. Sal, n = 229 neurons from 4 mice; coc, n = 259 neurons from 4 mice. p < 0.0001, 0.0001, 0.0001, two-tailed Mann-Whitney test for unpaired comparisons between saline and cocaine on each day. ***p <0.001. Data are presented as the mean ± s.e.m.. Source data are provided as a Source Data file.

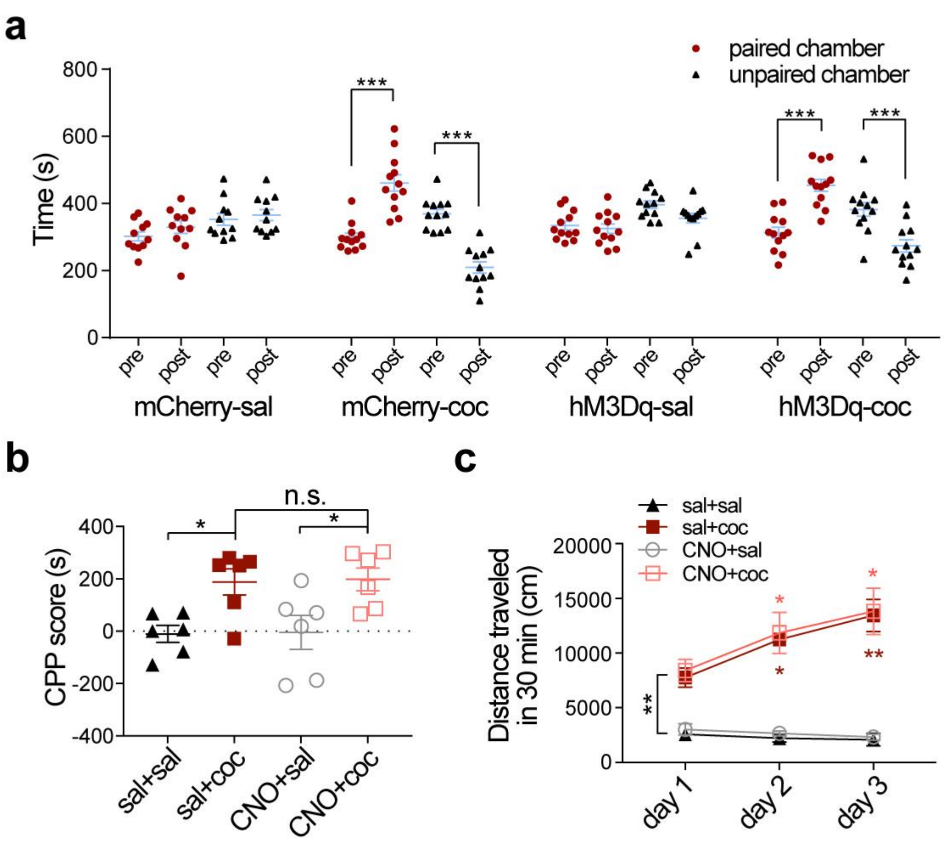

Fig. S5 (related to Fig. 1) Chemogenetic activation of FrA neurons fails to prevent cocaine-induced CPP. (**a**) The detailed data of Fig. 1m about the total time spent in each chamber during pretest and posttest which were used for calculating CPP scores. n = 11 (AAV-mCherry, sal), 12, 12, 12 mice. Paired two-tailed *t*-test for comparisons between pretest and posttest (marked in plot) and unpaired two-tailed *t*-test for comparisons between groups (n.s. for all comparisons between coc groups). (**b**) Statistics of CPP scores and (**c**) locomotor sensitization for intact C57BL/6J mice. CNO (0.5 mg/kg, i.p.) or saline control solution (sal) was injected 30 min before sal/coc (10 mg/kg, i.p.) following the protocol as in Fig. 1l. n = 6 mice per group. CPP: ordinary two-way ANOVA followed by Bonferroni's multiple comparisons test: coc effect, F (1, 20) = 16.6, p <0.001; CNO effect, F (1, 20) = 0.026, p = 0.87; interaction, F (1, 20) =

0.002, p = 0.97; multiple comparisons: p = 0.020, >0.99, = 0.017. Locomotor sensitization: in-group comparisons (between different training days) analyzed with one-way ANOVA followed by Dunnett's multiple comparisons: sal+coc, F (1.844, 9.221) = 16.72, p = 0.0010; CNO+coc, F (1.732, 8.662) = 9.579, p = 0.0075; multiple comparisons were performed for each cocaine group between day 2 or day 3 with day 1: p = 0.017, 0.0065 (sal+coc); p = 0.048, 0.025 (CNO+coc). Unpaired two-tailed *t*-test for comparisons between saline and cocaine groups on day 1, p = 0.0013, 0.0018. *p <0.05, **p <0.01. Data are presented as the mean ± s.e.m.. Source data are provided as a Source Data file.

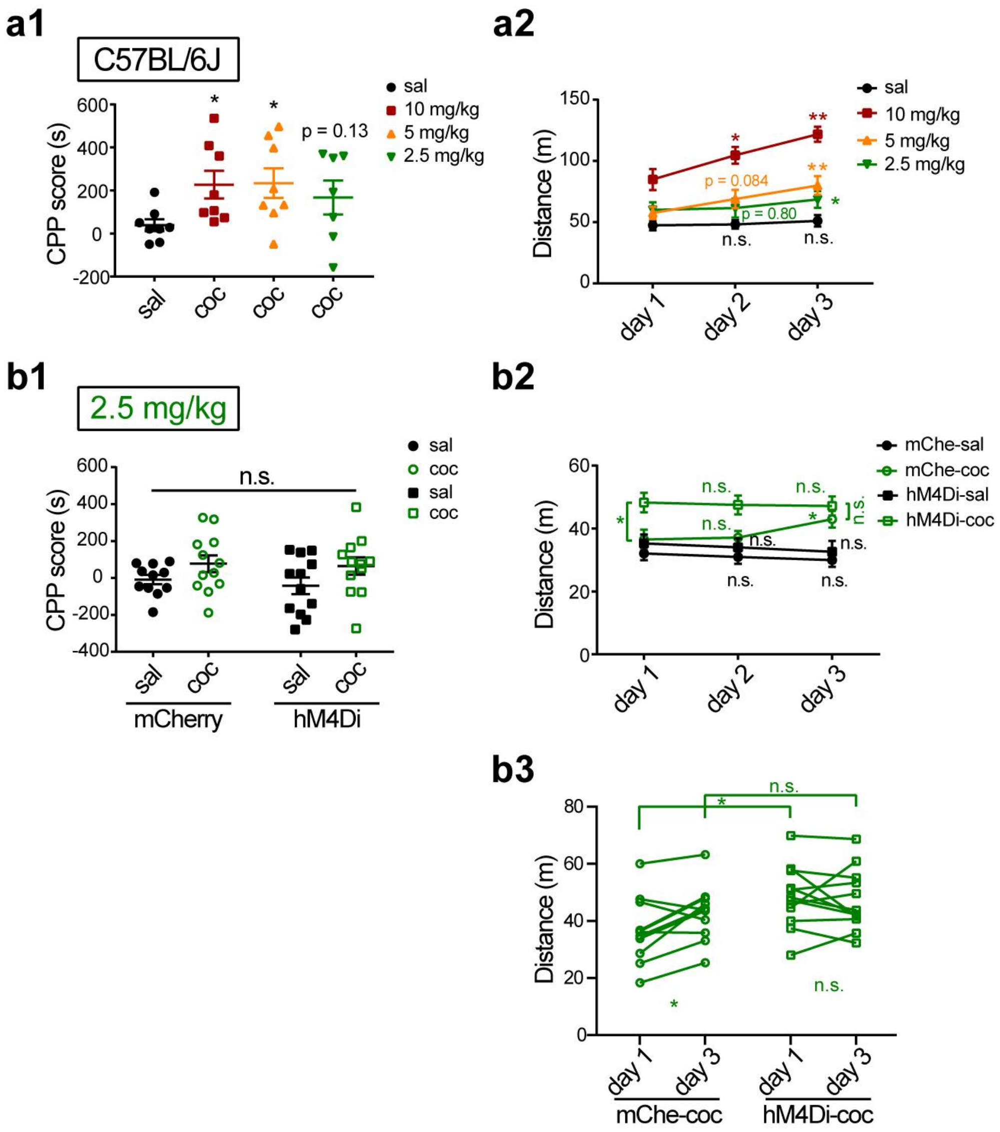

Fig. S6 (related to Fig. 1) Inhibitory modulation of FrA by hM4Di changes the subthreshold cocaine-induced locomotor sensitization. (**a**) Statistics of CPP scores (**a1**) and locomotor sensitization (**a2**) by different doses of cocaine (10, 5 or 2.5 mg/kg) for intact C57BL/6J mice, n = 8 mice per group. Unpaired two-tailed *t*-test for CPP scores comparisons between cocaine and saline groups, p = 0.018, 0.019, 0.13. In-group-comparisons (between different training days) of locomotor distance were analyzed with one-way ANOVA followed by Dunnett's multiple comparisons: sal, F

(1.996, 13.97) = 0.4961, p = 0.62; 10 mg/kg, F (1.176, 8.23) = 14.25, p = 0.0042; 5 mg/kg , F (1.748, 12.24) = 14.64, p = 0.0008; 2.5 mg/kg, F (1.773, 10.64) = 5.062, p = 0.032; multiple comparisons were performed for each group between day 2 or day 3 and day 1: saline, p = 0.97, 0.56; 10 mg/kg, p = 0.049, 0.0083; 5 mg/kg, p = 0.084, 0.0018; 2.5 mg/kg, p = 0.80, 0.030. Data are presented as the mean ± s.e.m.. (**b**) Statistics of CPP scores (**b1**) and locomotor sensitization (**b2**) by subthreshold dose of cocaine (2.5 mg/kg) for C57BL/6J mice with AAV-hM4Di (AAV-CaMKIIα-hM4Di-mCherry or AAV-CaMKIIα-mCherry as control) bilaterally expressed in FrA, n = 11 (mChe-sal), 12, 12, 12 mice. Detailed data points of day 1 and day 3 for cocaine groups are presented in (**b3**). CPP scores, ordinary two-way ANOVA followed by Bonferroni's multiple comparisons test: drug effect, F (1, 43) = 5.192, p = 0.028; virus effect, F (1, 43) = 0.302, p = 0.59; interaction, F (1, 43) = 0.05785, p = 0.81; all p values for multiple comparisons are not significant. Locomotor sensitization measurement, in-group comparisons (between different training days) were analyzed with one-way ANOVA followed by Dunnett's multiple comparisons: AAV-mCherry (sal), F (1.484, 14.84) = 0.9339, p = 0.39; AAV-mCherry (coc), F (1.336, 14.7) = 8.013, p = 0.0086; AAV-hM4Di (sal), F (1.91, 21.01) = 0.9565, p = 0.40; AAV-hM4Di (coc), F (1.647, 18.12) = 0.1479, p = 0.82; multiple comparisons were performed for each group between day 2 or day 3 and day 1: p = 0.79, 0.26; p = 0.94, 0.015; p = 0.71, 0.38; p = 0.91, 0.87. Unpaired two-tailed *t*-test for comparisons between cocaine groups on the day 1 and day 3: p = 0.015, 0.31. Data are presented as the mean ± s.e.m.. n.s., not significant; *p <0.05; **p <0.01. Source data are provided as a Source Data file.

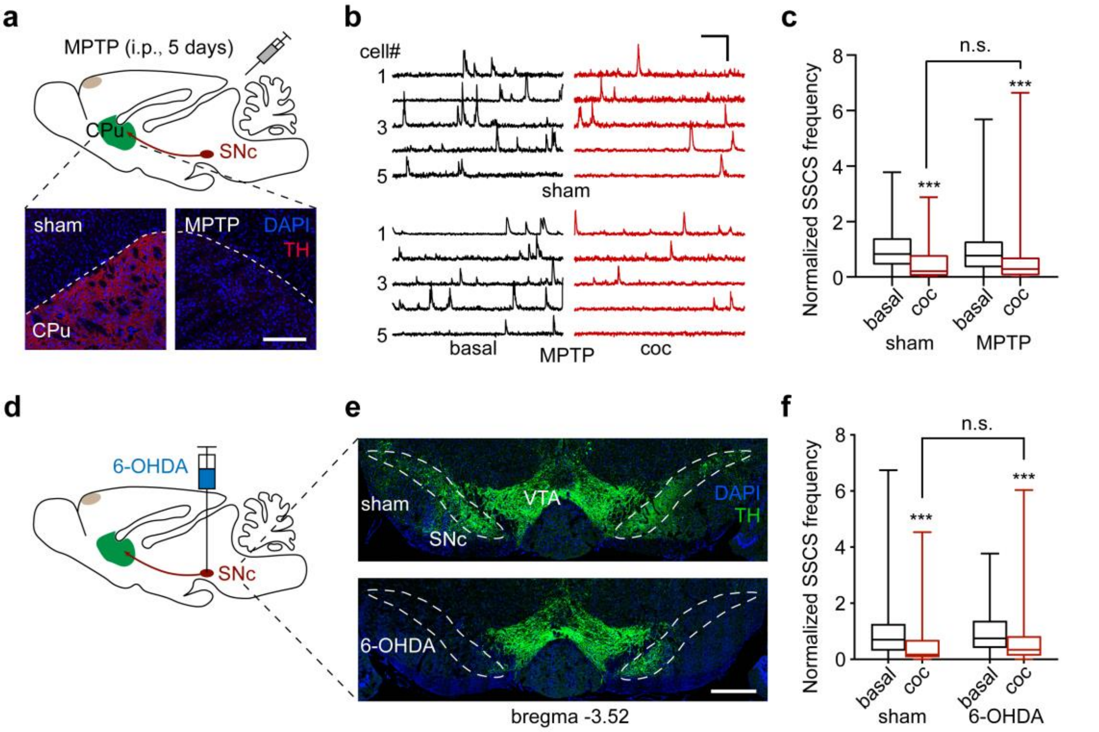

Fig. S7 (related to Fig. 2) SNc dopaminergic neurons are not necessary for cocaine-induced hypoactivity of the FrA. (**a**) Upper, dopaminergic SNc neurons lesioned by MPTP injection (30 mg/kg, i.p. daily for 5 days). Lower, TH-staining (red) showing the depletion of dopaminergic terminals in the CPu. The two micrographs were from the same region in the CPu of sham- and MPTP-treated mice and aligned symmetrically to the midline. AAV-CaMKIIα-GCaMP6s was used to monitor excitatory neurons in the FrA of C57BL/6J mice. Scale bar, 200 µm. (**b**, **c**) Representative traces and normalized SSCS frequency (minimum, maximum and 3 quartiles) of FrA pyramidal neurons in MPTP-treated mice. Sham, n = 227 neurons from 4 mice; MPTP, n = 196 neurons from 4 mice. Two-tailed Wilcoxon test for paired comparisons: p <0.0001, 0.0001; and two-tailed Mann-Whitney test for unpaired comparison: p = 0.96. Scale bars, 20 s, 300% dF/F_0. (**d, e**) Schematic of bilateral 6-OHDA injection into SNc and TH-staining (green) showing the loss of dopaminergic somata and fibers in SNc. Scale bar, 500 µm. (**f**)

Supplementary Information

Normalized SSCS frequency (minimum, maximum and 3 quartiles) of FrA neurons in 6-OHDA-treated mice. Sham, n = 168 neurons from 3 mice; 6-OHDA, n = 151 neurons from 3 mice. Two-tailed Wilcoxon test for paired comparisons: p <0.0001, 0.0001; and two-tailed Mann-Whitney test for unpaired comparison: p = 0.12. ***p <0.001. Source data are provided as a Source Data file.

Supplementary Information

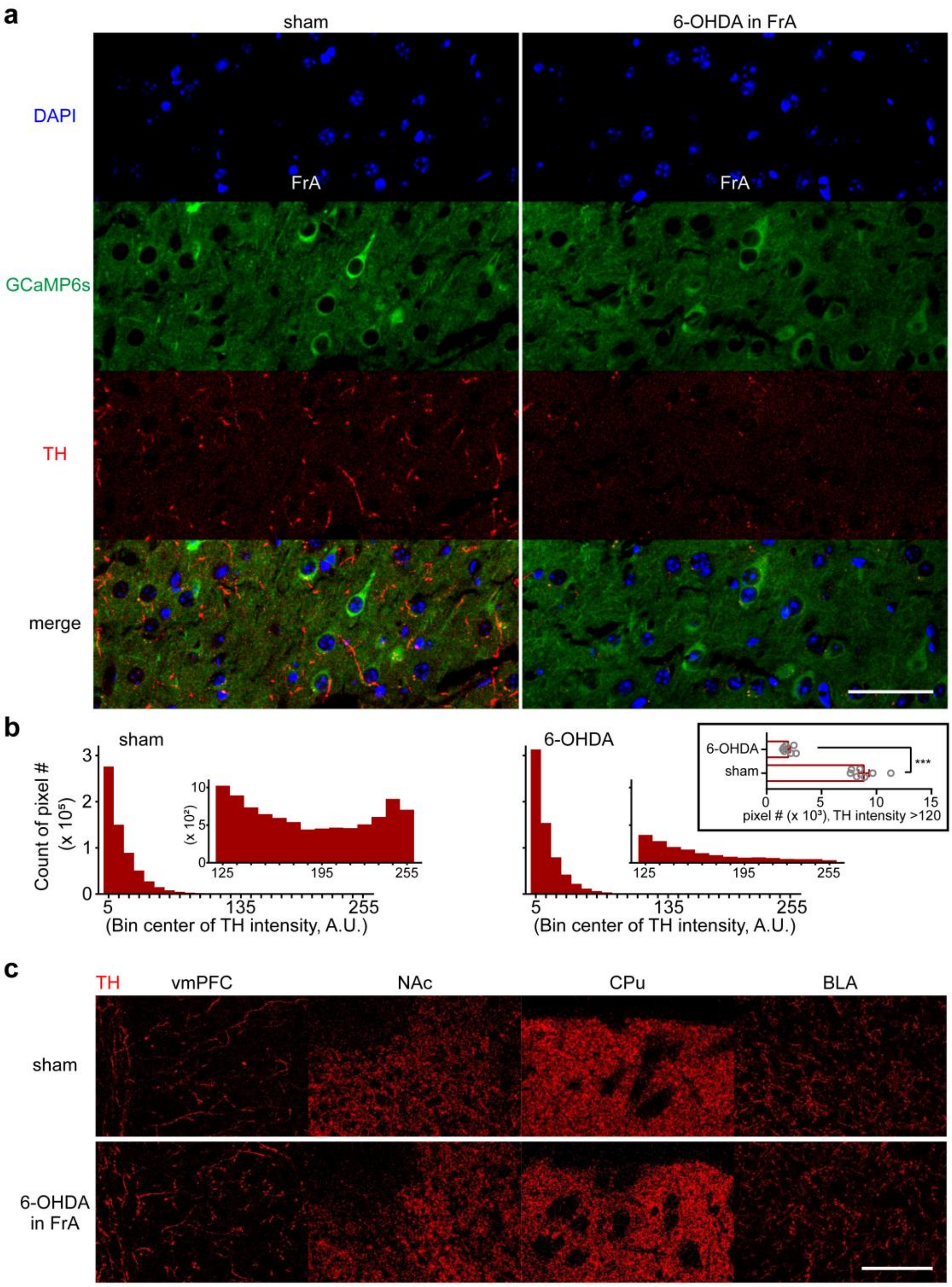

Fig. S8 (related to Fig. 3) Region-specific depletion of dopaminergic terminals following 6-OHDA injection in the FrA. (**a**) Representative micrographs (same as Fig. 3a) and (**b**) quantification showing the depletion of dopaminergic terminals in the FrA

with TH staining. Scale bar, 50 µm; pixel size, 0.208 µm; 1269*504 pixels. Boxed inset in (**b**) showing the comparison of pixel numbers (TH intensity >120 A.U.) between sham and 6-OHDA groups. n = 7 slices from 5 mice per group. Data are presented as the mean ± s.e.m.. p <0.0001, unpaired two-tailed *t*-test. (**c**) TH staining showing intact dopaminergic terminals in the vmPFC, NAc, CPu, and BLA following local injection of 6-OHDA into the FrA. Scale bar, 100 µm. Micrographs in (**a**) and (**c**) are from the same mouse. Similar results were obtained in 5 mice. Source data are provided as a Source Data file.

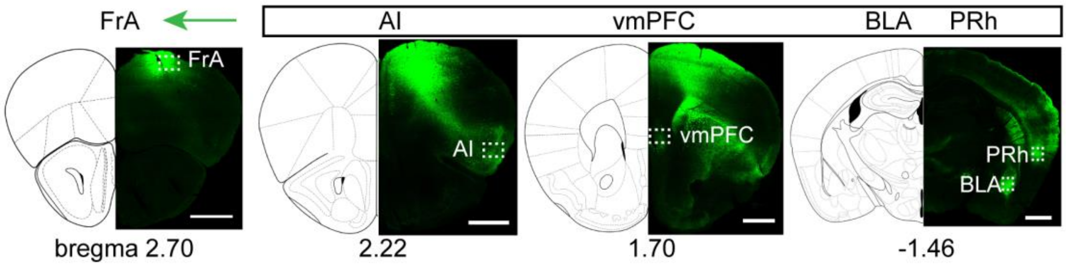

Fig. S9 (related to Fig. 3) Micrographs showing the retrograde-labeled brain regions upstream of the FrA. Representative micrographs of coronal slices showing 4 upstream regions. White dashed boxes indicate areas where somata are positively labeled by EYFP and presented in Fig. 3d. Scale bars, 1 mm.

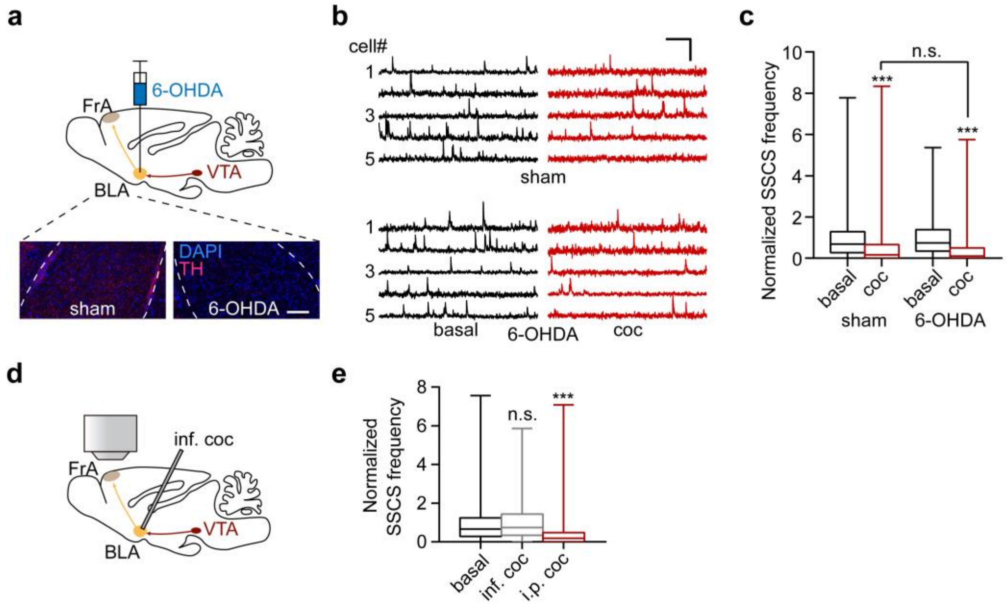

Fig. S10 (related to Fig. 4) Dopaminergic projections to the BLA are not necessary for cocaine-induced hypoactivity of the FrA. (**a**) Upper, schematic of 6-OHDA injection into the BLA. Lower, TH-staining (red) showing the depletion of dopaminergic terminals in the BLA through 6-OHDA microinjection. The two micrographs are from the same region of sham- or 6-OHDA-treated BLA and aligned symmetrically to the midline. Scale bar, 100 µm. (**b**, **c**) Representative traces and normalized SSCS frequency (minimum, maximum and 3 quartiles) of FrA neurons of sham- or 6-OHDA-treated mice before and after i.p. cocaine. Sham, n = 303 neurons from 5 mice; 6-OHDA, n = 245 neurons from 5 mice. Two-tailed Wilcoxon test for paired comparisons: p <0.0001, 0.0001; and two-tailed Mann-Whitney test for unpaired comparison: p = 0.19. Scale bars, 20 s, 200% dF/F_0. (**d**, **e**) Schematic of two-photon Ca^{2+} imaging and statistics (minimum, maximum and 3 quartiles) of normalized SSCS frequency of the FrA in response to cocaine microinfusion (inf. coc) into the BLA. n = 167 neurons from 4 mice. p = 0.59, <0.0001, two-tailed Wilcoxon test. ***p <0.001. Source data are provided as a Source Data file.

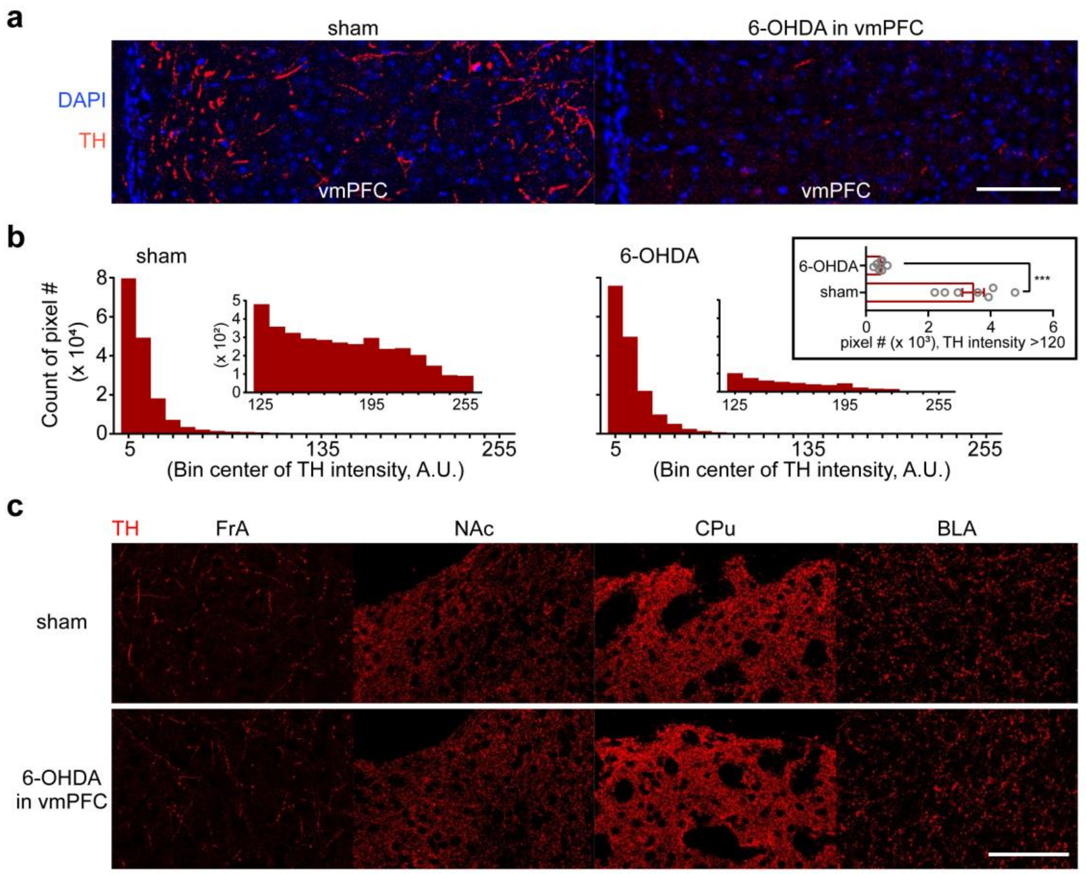

Fig. S11 (related to Fig. 4) Region-specific depletion of dopaminergic terminals following 6-OHDA injection into the vmPFC. (**a**) Representative micrographs (same as Fig. 4a) and (**b**) quantification showing the depletion of dopaminergic terminals in the vmPFC with TH staining. Scale bar, 100 μm; pixel size, 0.830 μm; 704*240 pixels. Boxed inset in (**b**) showing the comparison of pixel numbers (TH intensity >120 A.U.) between sham and 6-OHDA groups. n = 7 slices from 5 mice per group. Data are presented as the mean ± s.e.m.. p <0.0001, unpaired two-tailed *t*-test. (**c**) TH staining showing intact dopaminergic terminals in the FrA, NAc, CPu, and BLA following the local injection of 6-OHDA into the vmPFC. Micrographs in the sham or 6-OHDA group are from the same mouse. Similar results were obtained in 4 mice. Scale bar, 100 μm. Source data are provided as a Source Data file.

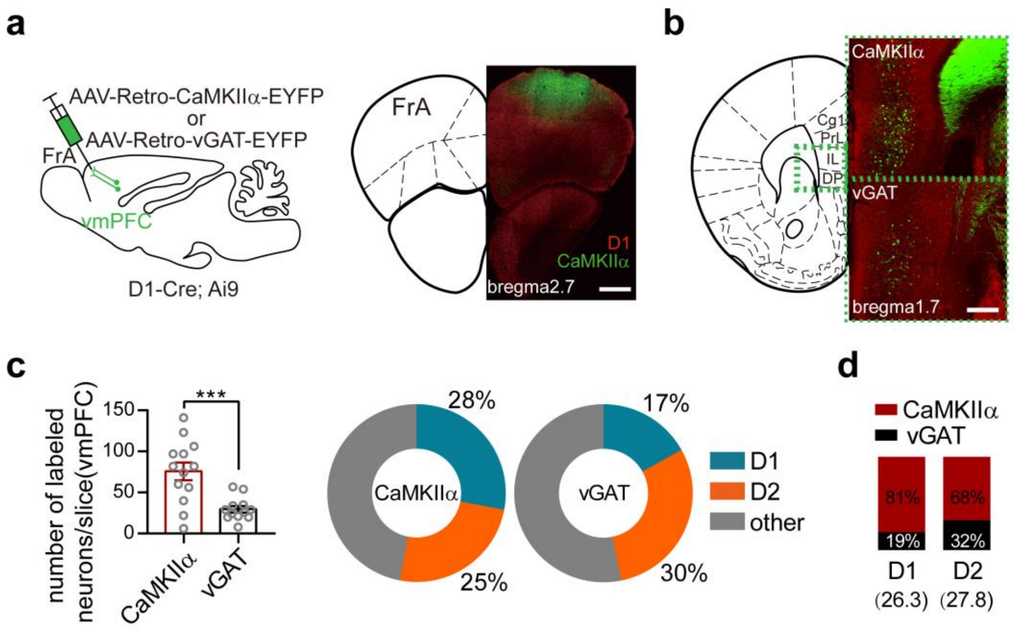

Fig. S12 (related to Fig. 5) Both vmPFC-D1R and -D2R neurons project to the FrA through excitatory and inhibitory innervations. (**a**) Schematic and representative micrographs showing retrograde virus injection into the FrA of D1-Cre and Ai9-crossed mice. Scale bar, 500 µm. (**b**) Representative micrographs of vmPFC slices showing labeled excitatory or inhibitory neurons (green). Scale bar, 200 µm. (**c**) Numbers (mean ± s.e.m.) of labeled excitatory and inhibitory neurons in the vmPFC and percentages of co-labeling with D1 or D2 receptors. Cells were counted in the vmPFC (IL and DP) in an area of 500×500 µm per slice. CaMKIIα, n = 13 slices from 2 D1- and 2 D2-mice; vGAT, n = 12 slices from 2 D1- and 2 D2-mice. p =0.0008, unpaired two-tailed *t*-test. ***p <0.001. (**d**) Percentage of excitatory and inhibitory neurons among FrA-projecting vmPFC-D1 or -D2 neurons, according to (**c**) without consideration of D1R & D2R that expressed in other types of neurons. Numbers in parentheses indicate the average numbers of FrA-projecting vmPFC-D1 or -D2 neurons in one slice within the defined area. Source data are provided as a Source Data file.

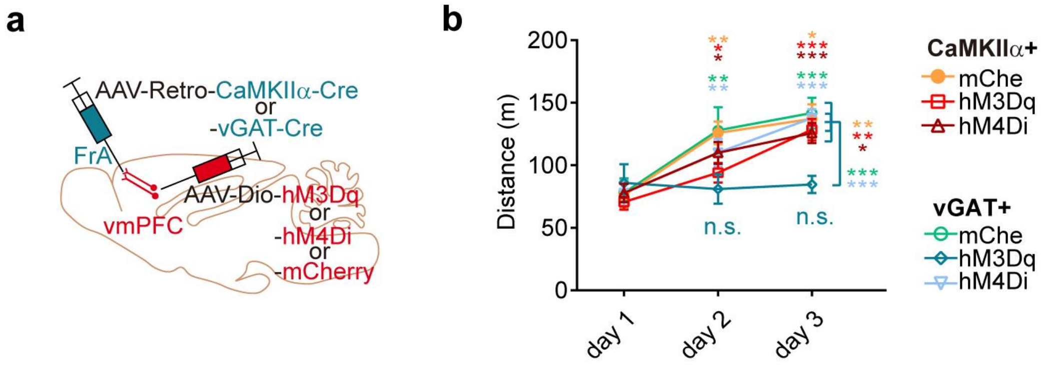

Fig. S13 (related to Fig. 6) Inhibitory vmPFC-FrA projections mediate cocaine-induced locomotor sensitization. (**a**) Schematic showing the virus injection and chemogenetic manipulation of FrA-projecting vmPFC excitatory or inhibitory neurons. Retrograde virus expressing Cre in FrA-projecting vmPFC excitatory or inhibitory neurons was injected bilaterally into the FrA and Cre-dependent Dio-virus expressing mCherry, hM3Dq or hM4Di was injected bilaterally into the vmPFC. (**b**) Statistics (mean ± s.e.m.) showing the cocaine-induced locomotor sensitization. The behavior paradigm is the same with Fig. 6. In-group comparisons (between different training days) were analyzed with one-way ANOVA followed by Dunnett's multiple comparisons: CaMKIIα+mChe, $F (1.116, 6.696) = 10.41$, $p = 0.014$; CaMKIIα+hM3Dq, $F (1.803, 12.62) = 29.05$, $p < 0.0001$; CaMKIIα+hM4Di, $F (1.76, 12.32) = 20.96$, $p = 0.0001$; vGAT+mChe, $F (1.734, 12.14) = 27.06$, $p < 0.0001$; vGAT+hM3Dq, $F (1.313, 9.194) = 0.09567$, $p = 0.83$; vGAT+hM4Di, $F (1.782, 12.47) = 32.12$, $p < 0.0001$; n = 7, 8, 8, 8, 8, 8 mice; multiple comparisons were performed for each group between day 2 or day 3 and day 1: p = 0.0018, 0.035; p = 0.022, 0.0006; p = 0.014, 0.0007; p = 0.0016, 0.0001; p = 0.91, 0.99; p = 0.0026, 0.0004. Comparisons between vGAT+hM3Dq and other 5 groups on day 3 were analyzed with one-way ANOVA followed by Dunnett's multiple comparisons: $F (5, 41) = 5.327$, $p = 0.0007$; multiple comparisons: p = 0.0015,

0.0067, 0.012, 0.0003, 0.0009. n.s., not significant; *$p < 0.05$; **$p < 0.01$; ***$p < 0.001$.

Source data are provided as a Source Data file.

Table S1. Statistical analysis in Fig. 1m, n.

**Fig 1m**	**Two-way ANOVA followed by Bonferroni's multiple comparisons**	
	Drug effect	F (1, 43) = 49.08, p <0.001
	Virus effect	F (1, 43) = 0.5337, p = 0.47
	Interaction	F (1, 43) = 1.364, p = 0.25
	mCherry: saline *versus* cocaine	p <0.0001
	hM3Dq: saline *versus* cocaine	p <0.0001
	saline: mCherry *versus* hM3Dq	P >0.99
	cocaine: mCherry *versus* hM3Dq	P >0.99
**Fig 1n**	**One-way ANOVA followed by Dunnett's multiple comparisons**	
	AAV-mCherry: cocaine effect across 3 days	F (1.921, 21.13) = 30.5, p <0.001
	AAV-mCherry (coc): day 2 *versus* day 1	p = 0.0023
	AAV-mCherry (coc): day 3 *versus* day 1	p <0.0001
	AAV-hM3Dq: cocaine effect across 3 days	F (1.406, 15.47) = 0.4257, p = 0.59
	AAV-hM3Dq (coc): day 2 *versus* day 1	p = 0.90
	AAV-hM3Dq (coc): day 3 *versus* day 1	p = 0.68
	**Two-way ANOVA followed by Bonferroni's multiple comparisons**	
	Day 1: drug effect	F (1, 43) = 61.52, p <0.0001
	Day 1: virus effect	F (1, 43) = 0.6946, p = 0.41
	Day 1: interaction	F (1, 43) = 0.3221, p = 0.57

	Day 1, mCherry: saline *versus* cocaine	$p < 0.0001$
	Day 1, hM3Dq: saline *versus* cocaine	$p < 0.0001$
**Fig 1n**	Day 1, saline: mCherry *versus* hM3Dq	$p > 0.99$
	Day 1, cocaine: mCherry *versus* hM3Dq	$p > 0.99$
	Day 3: drug effect	$F (1, 43) = 177.4$, $p < 0.0001$
	Day 3: virus effect	$F (1, 43) = 5.584$, $p = 0.023$
	Day 3: interaction	$F (1, 43) = 5.954$, $p = 0.019$
	Day 3, mCherry: saline *versus* cocaine	$p < 0.0001$
	Day 3, hM3Dq: saline *versus* cocaine	$p < 0.0001$
	Day 3, saline: mCherry *versus* hM3Dq	$p > 0.99$
	Day 3, cocaine: mCherry *versus* hM3Dq	$p = 0.0080$

Table S2. Statistical analysis in Fig. 6b, d, e.

**Fig 6b**	**Two-tailed Wilcoxon tests**	
	D1-mCherry: CNO *versus* basal	p = 0.27
	D1-mCherry: CNO+coc *versus* basal	p <0.0001
	D1-hM3Dq: CNO *versus* basal	p <0.0001
	D1-hM3Dq: CNO+coc *versus* basal	p <0.0001
	D1-hM4Di: CNO *versus* basal	p = 0.62
	D1-hM4Di: CNO+coc *versus* basal	p <0.0001
	D2-hM3Dq: CNO *versus* basal	p <0.0001
	D2-hM3Dq: CNO+coc *versus* basal	p <0.0001
	D2-hM4Di: CNO *versus* basal	p = 0.88
	D2-hM4Di: CNO+coc *versus* basal	p <0.0001
**Fig 6d**	**Two-tailed Wilcoxon tests**	
	(inf. sal + i.p. coc) *versus* basal	p <0.0001
	(inf. SKF + i.p. coc) *versus* basal	p <0.0001
	(inf. SCH + i.p. coc) *versus* basal	p = 0.55
	(inf. QP + i.p. coc) *versus* basal	p <0.0001
	(inf. sulp + i.p. coc) *versus* basal	p = 0.032
	**Two-tailed Mann-Whitney tests**	
	(inf. SKF + i.p. coc) *versus* (inf. sal + i.p. coc)	p = 0.0014

	(inf. SCH + i.p. coc) *versus* (inf. sal + i.p. coc)	p <0.0001
	(inf. QP + i.p. coc) *versus* (inf. sal + i.p. coc)	p <0.0001
**Fig 6d**	(inf. sulp + i.p. coc) *versus* (inf. sal + i.p. coc)	p = 0.0009
**Fig 6e1**	**Two-tailed paired *t*-test**	
	inf. sal + i.p. coc: post *versus* pre	p = 0.0060
	inf. SCH + i.p. coc: post *versus* pre	p = 0.012
	inf. sulp + i.p. coc: post *versus* pre	p = 0.010
	Two-tailed unpaired *t*-test	
	Posttest score: SCH *versus* sal	p = 0.064
	Posttest score: sulp *versus* sal	p = 0.49
**Fig 6e2**	**One-way ANOVA followed by Dunnett's multiple comparisons**	
	inf. sal: cocaine effect across 3 days	F (1.749, 10.5) = 17.66, p <0.001
	inf. sal + i.p. coc: day 2 *versus* day 1	p = 0.046
	inf. sal + i.p. coc: day 3 *versus* day 1	p = 0.0011
	inf. SCH: cocaine effect across 3 days	F (1.469, 8.812) = 15.08, p = 0.0022
	inf. SCH + i.p. coc: day 2 *versus* day 1	p = 0.12
	inf. SCH + i.p. coc: day 3 *versus* day 1	p = 0.0003
	inf. sulp: cocaine effect across 3 days	F (1.627, 11.39) = 4.407, p = 0.044
	inf. sulp + i.p. coc: day 2 *versus* day 1	p = 0.33
	inf. sulp + i.p. coc: day 3 *versus* day 1	p = 0.32

	**Unpaired two-tailed *t*-test**	
	Day 3: SCH *versus* saline	$p < 0.0001$
	Day 3: sulp *versus* saline	$p < 0.0001$
