# Supplementary material for: Cocaine induces locomotor sensitization through a dopamine-dependent VTA-mPFC-FrA cortico-cortical pathway in male mice
Source: Nat Commun. 2023 Mar 21;14:1568. doi: 10.1038/s41467-023-37045-3 (PMC10030897; doi:10.1038/s41467-023-37045-3)
Supplement: Supplementary file 3 — Reporting Summary [file 41467_2023_37045_MOESM3_ESM.pdf]

nature portfolio

Corresponding author(s): Zhuan Zhou

Last updated by author(s): Feb 3, 2023

Reporting Summary

Nature Portfolio wishes to improve the reproducibility of the work that we publish. This form provides structure for consistency and transparency in reporting. For further information on Nature Portfolio policies, see our Editorial Policies and the Editorial Policy Checklist.

Statistics

For all statistical analyses, confirm that the following items are present in the figure legend, table legend, main text, or Methods section.

n/a	Confirmed	
☐	☒	The exact sample size (*n*) for each experimental group/condition, given as a discrete number and unit of measurement
☐	☒	A statement on whether measurements were taken from distinct samples or whether the same sample was measured repeatedly
☐	☒	The statistical test(s) used AND whether they are one- or two-sided<br>*Only common tests should be described solely by name; describe more complex techniques in the Methods section.*
☒	☐	A description of all covariates tested
☐	☒	A description of any assumptions or corrections, such as tests of normality and adjustment for multiple comparisons
☐	☒	A full description of the statistical parameters including central tendency (e.g. means) or other basic estimates (e.g. regression coefficient) AND variation (e.g. standard deviation) or associated estimates of uncertainty (e.g. confidence intervals)
☐	☒	For null hypothesis testing, the test statistic (e.g. *F*, *t*, *r*) with confidence intervals, effect sizes, degrees of freedom and *P* value noted<br>*Give P values as exact values whenever suitable.*
☒	☐	For Bayesian analysis, information on the choice of priors and Markov chain Monte Carlo settings
☒	☐	For hierarchical and complex designs, identification of the appropriate level for tests and full reporting of outcomes
☒	☐	Estimates of effect sizes (e.g. Cohen's *d*, Pearson's *r*), indicating how they were calculated

Our web collection on statistics for biologists contains articles on many of the points above.

Software and code

Policy information about availability of computer code

Data collection	All of the softwares used for data collection are commercialized and specified in the manuscript, they are: FV1200MPE (Olympus), LabState5.10 (Anilab Software & Instruments), LSM710 (Carl Zeiss), MBA-1 (INBIO), Pulse (HEKA Elektronik).
Data analysis	Besides the commercial and open source softwares specified in the manuscript, we also used custom codes (built in MATLAB, 2016b) for data analysis. The custom codes can only be shared on request before its publication. Other softwares include: ImageJ (v1.51t, NIH, USA), Adobe Illustrator (v2019-23.1.0, Adobe Systems), IGOR Pro 6.37 (WaveMetrics), Prism 7 (GraphPad Software).

For manuscripts utilizing custom algorithms or software that are central to the research but not yet described in published literature, software must be made available to editors and reviewers. We strongly encourage code deposition in a community repository (e.g. GitHub). See the Nature Portfolio guidelines for submitting code & software for further information.

Data

Policy information about availability of data

All manuscripts must include a data availability statement. This statement should provide the following information, where applicable:

- Accession codes, unique identifiers, or web links for publicly available datasets
- A description of any restrictions on data availability
- For clinical datasets or third party data, please ensure that the statement adheres to our policy

All relevant data that support this study are available from the corresponding author upon reasonable request. The source data are provided as a Source Data file.

Human research participants

Policy information about studies involving human research participants and Sex and Gender in Research.

Reporting on sex and gender	N/A
Population characteristics	N/A
Recruitment	N/A
Ethics oversight	N/A

Note that full information on the approval of the study protocol must also be provided in the manuscript.

Field-specific reporting

Please select the one below that is the best fit for your research. If you are not sure, read the appropriate sections before making your selection.

☒ Life sciences ☐ Behavioural & social sciences ☐ Ecological, evolutionary & environmental sciences

For a reference copy of the document with all sections, see nature.com/documents/nr-reporting-summary-flat.pdf

Life sciences study design

All studies must disclose on these points even when the disclosure is negative.

Sample size	The sample size was not predetermined but similar to those in similar studies which were cited in the Statistical analysis section of Method.
Data exclusions	No data were excluded from the analyses.
Replication	All experiments were performed with at least three biological replicates
Randomization	Allocation was random.
Blinding	Investigators were not blinding to group allocation, because: 1) all the group allocations were random before data collection; 2) no data were excluded; 3) predetermined protocols, custom codes and softwares were used for all data collection and analysis, whether blinding or not was not relevant to the results.

Reporting for specific materials, systems and methods

We require information from authors about some types of materials, experimental systems and methods used in many studies. Here, indicate whether each material, system or method listed is relevant to your study. If you are not sure if a list item applies to your research, read the appropriate section before selecting a response.

Materials & experimental systems

n/a	Involved in the study	
☐	☒	Antibodies
☒	☐	Eukaryotic cell lines
☒	☐	Palaeontology and archaeology
☐	☒	Animals and other organisms
☒	☐	Clinical data
☒	☐	Dual use research of concern

Methods

n/a	Involved in the study	
☒	☐	ChIP-seq
☒	☐	Flow cytometry
☒	☐	MRI-based neuroimaging

Antibodies

Antibodies used	1) rabbit anti-TH primary antibody (AB152, Millipore);<br>2) mouse anti-GAD67 primary antibody (MAB5406, Millipore);<br>3) secondary antibodies (donkey anti-rabbit 594, A21207; donkey anti-mouse 488, A21202; Invitrogen).
Validation	1) Validation statements on the manufacturer's website: "Anti-Tyrosine Hydroxylase Antibody detects level of TH and has been published and validated for use in ELISA, IF, IH, IH(P), IP and WB. It is expected that the antibody will react with most mammalian and

many non-mammalian species. It has been reported that this antibody does not work on paraffin embedded human tissue." (https://www.emdmillipore.com/US/en/product/Anti-Tyrosine-Hydroxylase-Antibody,MM_NF-AB152?bd=1#overview)
2) Validation statements on the manufacturer's website: "Reacts with the 67kDa isoform of Glutamate Decarboxylase (GAD67) of rat, mouse and human origins, other species not yet tested. This Anti-GAD Antibody, clone 1G10.2 is validated for use in IH, IH(P), WB for the detection of GAD67." (https://www.emdmillipore.com/US/en/product/Anti-GAD67-Antibody-clone-1G10.2,MM_NF-MAB5406?bd=1#overview)

Animals and other research organisms

Policy information about studies involving animals; ARRIVE guidelines recommended for reporting animal research, and Sex and Gender in Research

Laboratory animals	Adult (3–4 months old) male mice were used in this study, strains including: C57BL/6J, vGAT-Cre, DAT-Cre, DAT-KO, DAT-CI, D1-Cre (Drd1a-Cre), D2-Cre (Drd2-Cre), and Ai9.
Wild animals	The study did not involve wild animals.
Reporting on sex	Only male mice were used for this study.
Field-collected samples	This study did not involve samples collected from the field.
Ethics oversight	The use and care of animals were approved and directed by the Animal Care and Use Committee of Peking University and the Association for Assessment and Accreditation of Laboratory Animal Care (AAALAC).

Note that full information on the approval of the study protocol must also be provided in the manuscript.
